# Isoform Switch of Pyruvate Kinase M1 Indeed Occurs but Not to Pyruvate Kinase M2 in Human Tumorigenesis

**DOI:** 10.1371/journal.pone.0118663

**Published:** 2015-03-04

**Authors:** Cheng Zhan, Li Yan, Lin Wang, Jun Ma, Wei Jiang, Yongxing Zhang, Yu Shi, Qun Wang

**Affiliations:** 1 Department of Thoracic Surgery, Zhongshan Hospital, Fudan University, Shanghai, China; 2 Department of Radiation Oncology, Eye & ENT Hospital, Fudan University, Shanghai, China; Colorado State University, UNITED STATES

## Abstract

Muscle type of pyruvate kinase (PKM) is one of the key mediators of the Warburg effect and tumor metabolism. Due to alternative splicing, there are at least 12 known isoforms of the PKM gene, of which PKM1 and PKM2 are two major isoforms with only a 23 amino acid sequenced difference but quite different characteristics and functions. It was previously thought the isoform switch from PKM1 to PKM2 resulted in high PKM2 expression in tumors, providing a great advantage to tumor cells. However, this traditional view was challenged by two recent studies; one study claimed that this isoform switch does not occur during the Warburg effect; the other study asserted that the isoform switch is tissue-specific. Here, we re-analyzed the RNA sequencing data of 25 types of human tumors from The Cancer Genome Atlas Data Portal, and confirmed that PKM2 was the major isoform in the tumors and was highly elevated in addition to the entire PKM gene. We further demonstrated that the expression level of PKM1 significantly declined even though there was substantially increased expression of the entire PKM gene. The proportion of PKM1 in total transcript variants also significantly declined in tumors but the proportion of PKM2 did not change accordingly. Therefore, we conclude that the isoform switch of PKM1 does indeed occur, but it switches to other isoforms rather than PKM2. Considering the change in the expression levels of PKM1, PKM2 and the entire PKM gene, we propose that the upregulation of PKM2 is primarily due to elevated transcriptional levels of the entire PKM gene, instead of the isoform switch.

## Introduction

Numerous metabolic changes during tumorigenesis have received increased attention in recent years. The best known and probably most central change is the unusually high rate of glycolysis and lactate production, which was originally described by Otto Warburg in the 1920s, and was subsequently termed the Warburg effect. Although the mechanisms underlying the Warburg effect are not completely understood, it is widely thought that muscle type of pyruvate kinase (PKM) plays a key role.

The PKM gene produces two major alternatively spliced isoforms, PKM1, which is constitutively active, and PKM2, which can switch between an active tetrameric and more common inactive dimerform. PKM2 is highly upregulated in cancer cells, and the dynamic tuning of its activity causes the transition from aerobic respiration to glycolysis. This transition is customarily believed to result in the accumulation of phosphoenolpyruvate and other glycolytic intermediates, and consequently, the redirection of glucose for macromolecule iosynthesis rather than ATP production, which provides a great advantage for the growth and division of cancer cells. Currently, there are 10 other isoforms of the PKM gene recorded in the University of California Santa Cruz (UCSC) Genome Bioinformatics database (http://genome.ucsc.edu), but their characteristics, functions, and expression have received little attention and remain unclear.

For decades, it was widely believed that PKM1 was specific for non-proliferating tissues and PKM2 for proliferating tissues, and that the isoform switch from PKM1 to PKM2 was the main reason for increased PKM2 expression in cancers [1–3]. However, in 2011, Bluemlein *et al*. [4] demonstrated that PKM2 was not specific for tumors. In addition, she found no evidence of a PKM1 to PKM2 isoform shift during tumorigenesis [4]. In 2013 Desai *et al*. [5] claimed that the isoform switch from PKM1 to PKM2 only occurred in glioblastomas, and not in other tumor types based on The Cancer Genome Atlas (TCGA) data analysis. With the exception of the two aforementioned studies, there is no additional evidence on whether the PKM1 to PKM2 isoform switch occurs. Here, we reanalyzed data generated by the TCGA Research Network, and concluded that a PKM1 isoform switch did indeed occur, but it switched to other isoforms rather than PKM2. Instead, the upregulation of PKM2 in human tumors was primarily due to elevated transcriptional levels of the entire gene.

## Material and Methods

### Data acquisition and processing

Level 3 RNA sequencing (RNA-Seq) V2 data (contains data on gene, isoform, exon, and junction levels), which was released by TCGA before April 15, 2014, were downloaded from the TCGA data portal (https://tcga-data.nci.nih.gov/tcga/tcgaHome2.jsp), and included 618 human normal samples and 7,290 tumor samples from 25 tissue types (Table 1). Fourteen transcript variants of PKM were identified from TCGA data, including uc002atr.1, uc002ats.1, uc002att.1, uc002atu.1, uc002atv.1, uc002atw.1, uc002atx.1, uc002aty.1, uc002atz.1, uc010bit.1, uc010biu.1, uc010uki.1, uc010ukj.1, and uc010ukk.1. The sequences of these transcript variants and their protein products were obtained from UCSC Genome Bioinformatics. These sequences were compared to the PKM gene sequence recorded in the NCBI Gene database (http://www.ncbi.nlm.nih.gov/gene) using BLAST to determine the transcriptional patterns of these 14 transcript variants. Finally, we assigned uc002atw.1 and uc002atx.1 to PKM1, and uc002aty.1 to PKM2. RNA-Seq by expectation maximization (RSEM) values were used to represent the expression levels of these transcript variants. Data were presented as mean ± standard error of mean (SEM).

**Table 1:** The number of normal samples and tumor samples in each tissue type.

Tissue Type	Normal	Tumor
Acute Myeloid Leukemia (LAML)	0	173
Adrenocortical carcinoma (ACC)	0	79
Bladder Urothelial Carcinoma (BLCA)	19	241
Brain Lower Grade Glioma (LGG)	0	469
Breast invasive carcinoma (BRCA)	110	1034
Cervical squamous cell carcinoma and endocervical adenocarcinoma (CESC)	3	196
Colon adenocarcinoma (COAD)	41	262
Glioblastoma multiforme (GBM)	0	169
Head and Neck squamous cell carcinoma (HNSC)	43	498
Kidney Chromophobe (KICH)	25	66
Kidney renal clear cell carcinoma (KIRC)	72	519
Kidney renal papillary cell carcinoma (KIRP)	30	198
Liver hepatocellular carcinoma (LIHC)	50	191
Lung adenocarcinoma (LUAD)	58	490
Lung squamous cell carcinoma (LUSC)	50	490
Lymphoid Neoplasm Diffuse Large B-cell Lymphoma (DLBC)	0	28
Ovarian serous cystadenocarcinoma (OV)	0	266
Pancreatic adenocarcinoma (PAAD)	3	85
Prostate adenocarcinoma (PRAD)	50	333
Rectum adenocarcinoma (READ)	9	164
Sarcoma (SARC)	2	105
Skin Cutaneous Melanoma (SKCM)	1	373
Thyroid carcinoma (THCA)	29	275
Uterine Carcinosarcoma (UCS)	0	57
Uterine Corpus Endometrial Carcinoma (UCES)	23	529
Total	618	7290

### Statistical analysis

Data were analyzed using IBM SPSS for Windows, Version 20 (Armonk, NY, USA). Student’s *t*-test was used to evaluate the expression differences and Mann-Whitney U test was used to evaluate the percentage differences of PKM isoforms between normal and tumor samples.

## Results

### Transcript variants and isoforms of PKM

**Fig 1:**
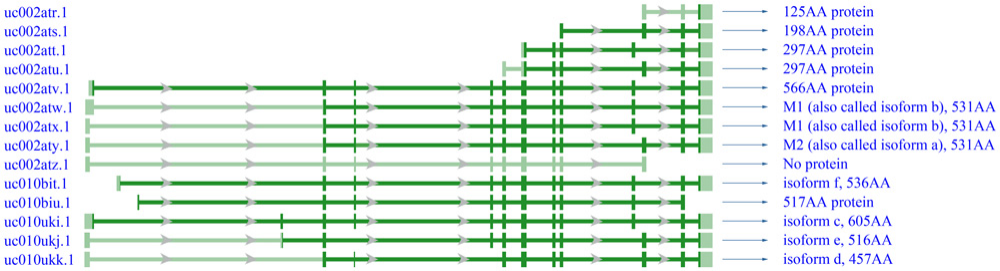
Expression patterns of the 14 transcript variants of PKM. Wide bars represent exons and narrow horizontal lines represent introns. The dark green color represents the sequence between the translation initiation codon and termination codon, whereas the light green color represents the sequence outside the codons. The arrows point in the 5′ to 3′ direction. AA: amino acid.

To study the expression profiles of PKM and its isoforms in human tumors, we analyzed TCGA Level 3 expression data from samples that were profiled using RNA-Seq technology, and identified 14 transcript variants of PKM. PKM1, PKM2, and 10 other known isoforms are translated from these transcript variants. The transcriptional patterns of these transcript variants are shown in Fig. 1. Of these transcript variants, uc002atw.1 and uc002atx.1 are translated to PKM1, and uc002aty.1 is translated to PKM2. PKM1 and PKM2 only differ by 23 amino acid residues within a 56-residue alternatively spliced exon.

In Desai et al. [5], the authors confused the transcript variants of PKM, and incorrectly assigned uc002att.1, uc002atv.1, uc002atw.1, uc002atx.1, uc010ukj.1, uc010ukk.1, uc010bit.1 to PKM1, and uc002atr.1, uc002ats.1, uc002atu.1, uc002aty.1, uc010biu.1, uc010uki.1, uc002atz.1 to PKM2. This mistake resulted in their inaccurate results and conclusions.

The RSEM value of each transcript variant was calculated, and the results are summarized in S1 and S2 Tables. The number of normal and tumor samples, and the expression levels of each transcript variant of PKM in each tissue type are also shown.

### PKM expression is highly elevated in human tumors

**Fig 2:**
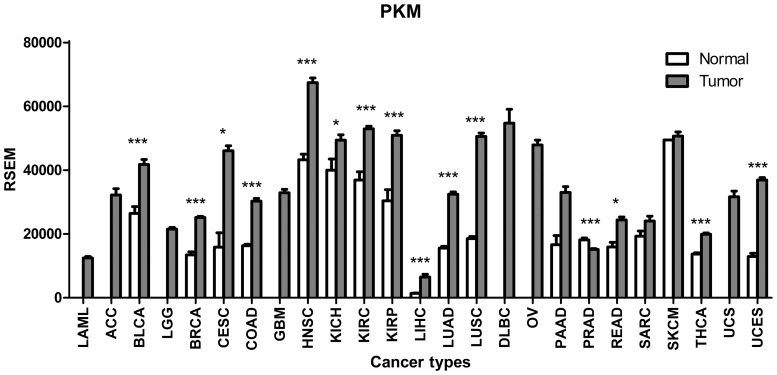
Expression profile of PKM in human tumors. PKM is a highly expressed gene and is elevated in most human tumors. *p<0.05, **p<0.01, ***p<0.001.

As shown in Fig. 2 and S3 Table, PKM is a highly expressed gene with a RSEM value of more than 10,000 in both normal and tumor tissues of each organ, except the liver. In addition, PKM expression is elevated in most human tumors. The results of our statistical analyses showed that in 18 kinds of tumors with normal sample controls available, PKM expression significantly increased in 14 tumor types and significantly decreased in only one tumor type, whereas in the other three tumor types, the difference in expression between the tumor and control samples was not statistically significant.

### PKM2 expression increases in human tumors, but its proportion does not change

**Fig 3:**
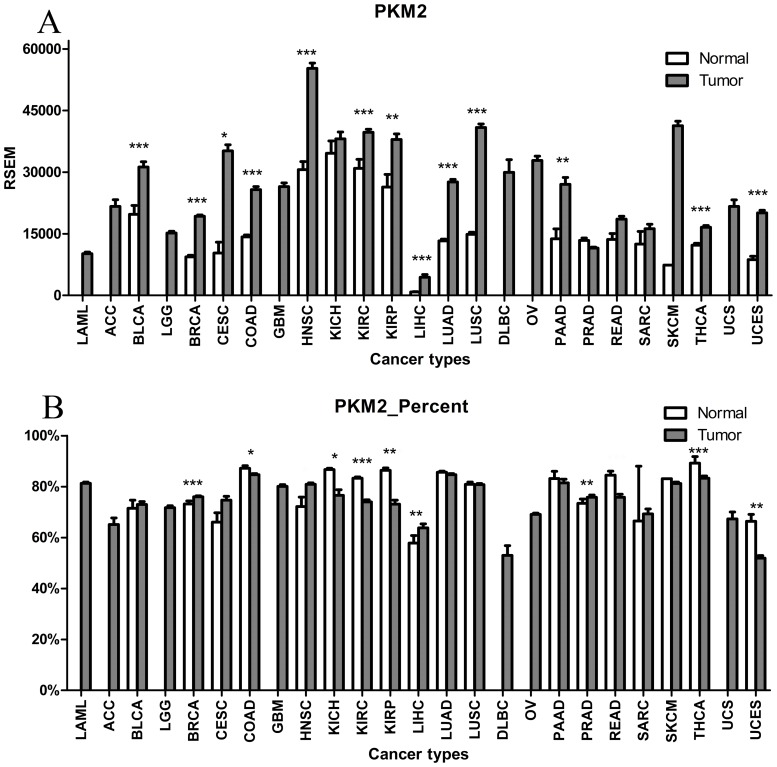
Expression and proportion of PKM2 in human tumors. (A) Expression of PKM2; (B) Proportion of PKM2. Expression of PKM2 increased, but the proportion did not change in human tumors.

As shown in Fig. 3 and S3 Table, PKM2 expression significantly increased in 13 tumor types, and did not significantly decrease in any of the tumors, similar to PKM. PKM2 was the major isoform identified, and accounted for 51.9–87.2% of all isoforms in both normal and tumor samples in the 25 tissue types. Although PKM2 expression significantly increased in most tumor types, the percentage of PKM2 in the total transcript variants did not significantly change. The results of the statistical analyses showed that the proportion of PKM2 significantly increased in three tumor types, and decreased in six cancer types, whereas in the other nine cancer types the difference in expression was not statistically significant.

### Expression and proportion of PKM1 both decrease in human tumors

**Fig 4:**
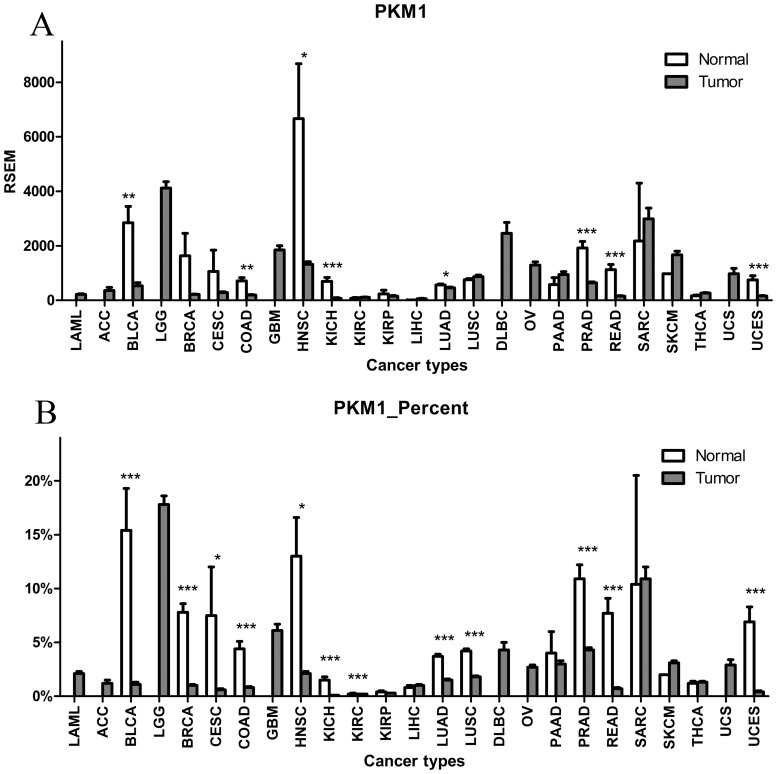
Expression and proportion of PKM1 in human tumors. (A) PKM1 expression; (B) proportion of PKM1. Expression and proportion of PKM1 both decreased in human tumors.

As shown in Fig. 4 and S3 Table, PKM1 only accounted for a small proportion of total transcripts, ranging from 0.1% to 17.8%, which was much lower than the amount of PKM2, even in normal tissues. PKM1 expression significantly decreased in eight tumor types, and did not significantly increase in any tumor type. Taking into account the increased expression of the entire PKM gene, the percentage of PKM1 decreased more significantly in tumors compared with normal control samples. The percentage of PKM1 in total transcript variants significantly decreased in 11 tumor types and only significantly increase in one tumor type.

### Increase in PKM2 expression is much greater than the decrease in PKM1 in human tumors

**Fig 5:**
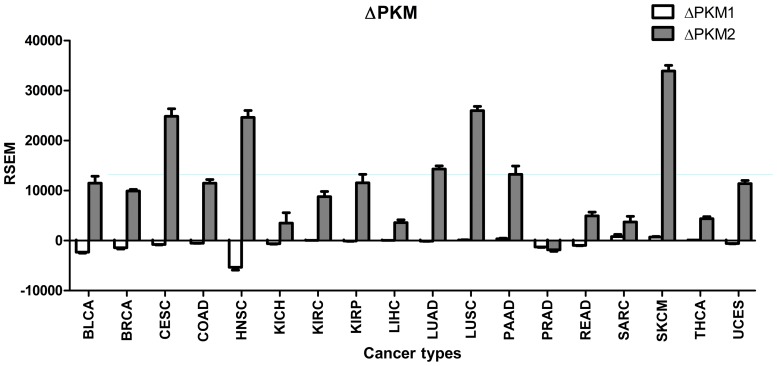
Change in expression of PKM1 and PKM2 in human tumors compared with normal tissues. There was little change in PKM1 expression, whereas there was a significant increase in PKM2 in human tumors.

As shown in Fig. 5, PKM1 expression only changed by several hundred RSEM, whereas PKM2 expression increased by RSEM values of tens of thousands in most tumor types. The change in PKM2 expression was 4.5- to 200-fold greater than that of PKM1 in all human tumors, except prostate adenocarcinoma. Thus, we postulated that the upregulation of PKM2 in human tumors compared with normal control samples is not due to changes in PKM1 levels.

## Discussion

In this study, we examined the mRNA profile of PKM in many types of human tumors based on TCGA data. We discovered that the expression level of PKM1 significantly declined even though there was substantially increased expression of the entire PKM gene in most human tumors. Both the expression and proportion of PKM1 declined, indicating that the PKM1 isoform switch did indeed occur. However, the proportion of PKM2 did not increase as PKM1 levels decreased. We found that the isoforms whose proportions were most increased were uc002atr.1 and uc002ats.1 (S1 and S2 Tables), rather than PKM2. Therefore, we postulated that PKM1 isoform switch did occur, but not to PKM2 in human tumors. Instead, it appeared that the upregulation of PKM2 was primarily due to the elevated transcriptional level of the entire PKM gene, because the change in PKM2 was consistent with PKM, and was far greater than the change in PKM1 expression levels.

Previous studies reported that PKM1 dominated in normal tissues and its dominance was replaced by PKM2 in cancer development [1–3]. In fact, these results were mostly derived by Western blot or immunohistochemistry, both of which were based on the reaction of antigen and antibody in protein level, unable to accurately quantify the expression of PK isoforms or distinguish their dominances. In a new research, Wilhelm *et al*. [6] discovered that the mRNA/protein ratio was remarkably conserved for each protein, and the actual amount of protein was primarily controlled by mRNA levels; therefore, the protein abundance of the PKM isoforms were supposed to be consistent with their mRNA profiles analyzed in our investigation. Now it is no doubted in the tremendous elevation of PKM2 in tumors, and Christofk *et al*. [2] have demonstrated that the elevated PK2 expression was necessary for aerobic glycolysis and provided a selective growth advantage for tumor cells. In Bluemlein *et al*. [4], the authors demonstrated for the first time that the PKM2 protein was the dominant isoform of PKM by quantitative mass spectrometry in both normal and tumor tissues and this finding was validated by our analysis of the TCGA RNA-Seq data In our research, we confirmed that PKM1 significantly declined in many types of tumors, which was consistent with previous studies but not the results of Bluemlein *et al* [2–4]. Due to the small sample number and low levels of PKM1 expression, the study by Bluemlein *et al*. [4] might not have been sensitive enough to detect the dip in PKM1 expression. Considering the change of PKM, PKM1 and PKM2, We believed the elevation of PKM2 was primarily due to the elevation of the entire PKM gene, instead of the switch of PKM1.

In the study by Desai *et al*. [5], the authors confused the isoforms of PKM, so what they thought was “PKM1” was actually a set of PKM isoforms, and similarly for “PKM2”. This may lead to that their conclusion differs from ours. With the exception of PKM1 and PKM2, the characteristics and functions of the other isoforms remain unclear.

We noticed that prostate adenocarcinoma was the only cancer type in which PKM significantly decreased compared with the normal controls, perhaps due to the slow growing nature of this cancer [7]. In addition, there was lower expression of PKM in liver tissues and hepatocellular carcinomas, probably because of the high proportion of liver and red blood cell type of pyruvate kinase (PKLR, 80.6% in normal liver samples, 46.4% in hepatocellular carcinomas, and less than 1% in most other samples) (S1 Fig.).

We observed that many other glycolytic enzymes, such as hexokinase 2 (HK2), phosphoglycerate kinase 1 (PGK1), glyceraldehyde-3-phosphate dehydrogenase (GAPDH), and lactate dehydrogenase A (LDHA) were increased in most tumors in the TCGA data portal (S2 Fig.), consistent with numerous studies [8–20]. In addition, there was a strong positive correlation with the expression of these glycolytic enzymes. Therefore, these glycolytic enzymes may be regulated by tumorigenic factors or may regulate tumorigenesis in parallel. For one example, many glycolytic enzymes are highly correlated with tumor prognosis [5,12–28]; for another example, hypoxia inducible factor-1 (HIF-1) is one of the key regulators of many glycolytic enzymes and of the Warburg effect [13,21,29–35].

For the past few years, PKM2 was found to not only act as a key enzyme of glycolysis in cytoplasm, but also can be translocated into the nucleus of tumor cells via many different mechanisms [36,37]. The nuclear PKM2 interacts with a lot of molecules important in regulating gene transcription and promoting cell proliferation, such as nuclear histone 1, signal transducers and activators of transcription 3 (STAT3), phosphorylates prothymosin a, b-catenin, HIF-1, and octamer-binding transcription factor 4 (Oct-4) [38–45]. Some of these interactions were believed to be caused by the protein kinase activity of PKM2 [38,42]. In addition, GAPDH and aldolase A undergo nuclear translocation in some cases, and promote cell proliferation or apoptosis, too [46,47].

In recent years, RNA-Seq has emerged as a popular and powerful tool for high throughput whole genome analysis, including transcript quantification, differential expression testing, reference-based gene annotation, and *de novo* transcript assembly. Using this technique, transcript levels are usually normalized by RSEM or reads per kilobases per million mapped reads (RPKM), which ideally facilitate the transparent comparison between multiple genes and samples [48,49]. As such, RNA-Seq will play an increasingly important role in future research studies.

## Supporting Information

S1 Fig Expression of PKLR and proportion of PKLR in PKM and PKLR in human tumors. (TIF)

S2 Fig Expression of HK2, GAPDH, PGK1 and LDHA in human tumors. (TIF)

S1 Table Expression and proportion of 14 PKM transcript variants in normal tissue samples. Transcript variants uc002atw.1 and uc002atx.1 are translated to M1; uc002aty.1 is translated to M2. Data are presented by mean and SEM.
(DOC)

S2 Table Expression and proportion of 14 PKM transcript variants in tumor tissue samples. (DOC)

S3 Table Expression and proportion of PKM1, PKM2, and PKM. (DOC)
