# Supplementary figures and images for: Isoform Switch of Pyruvate Kinase M1 Indeed Occurs but Not to Pyruvate Kinase M2 in Human Tumorigenesis
Source: PLoS One. 2015 Mar 4;10(3):e0118663. doi: 10.1371/journal.pone.0118663 (PMC4349452; doi:10.1371/journal.pone.0118663)

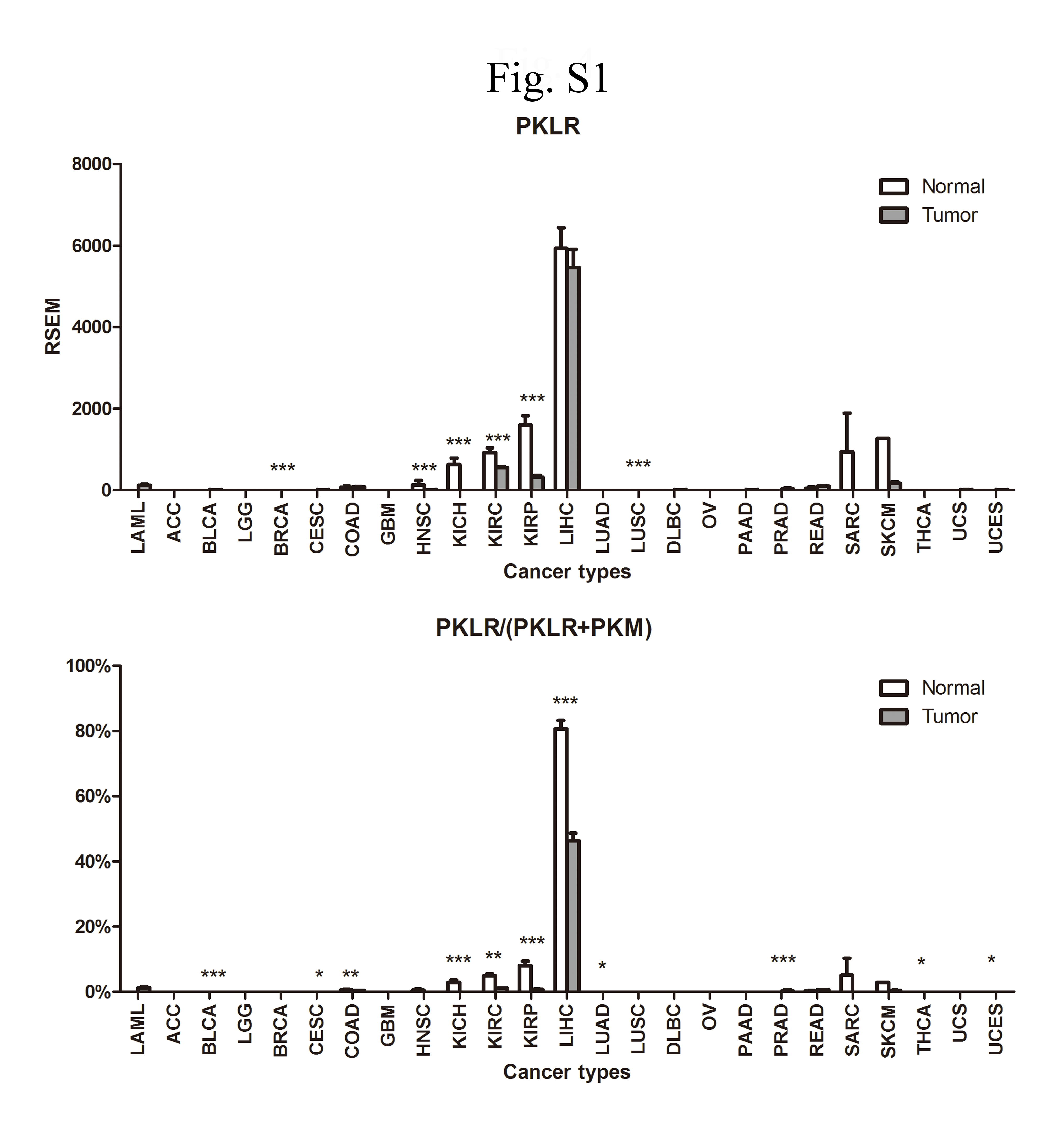

Supplement: S1 Fig — (TIF) [file pone.0118663.s001.tif]

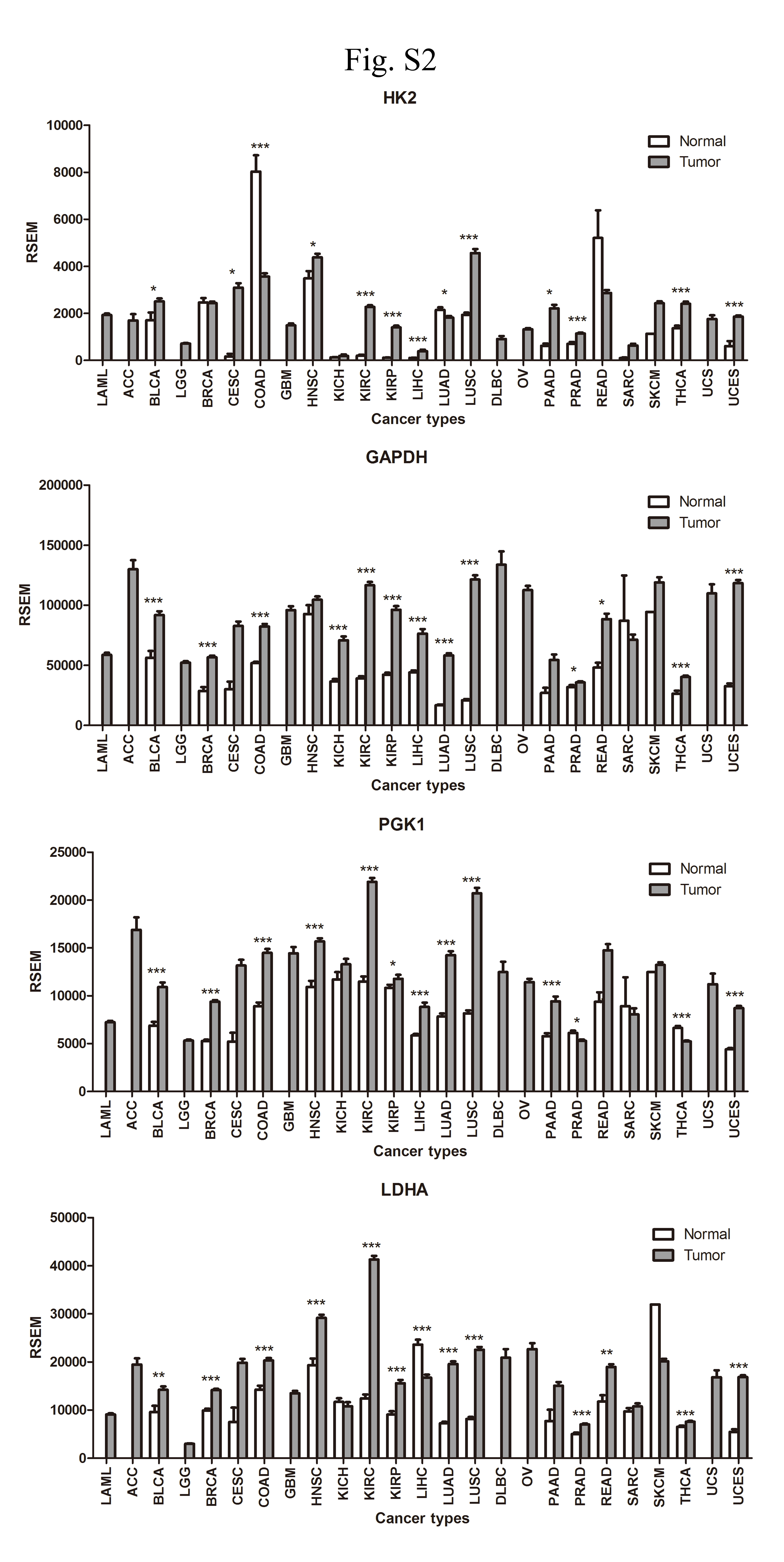

Supplement: S2 Fig — (TIF) [file pone.0118663.s002.tif]
