# Supplementary material for: Isoform Switch of Pyruvate Kinase M1 Indeed Occurs but Not to Pyruvate Kinase M2 in Human Tumorigenesis
Source: PLoS One. 2015 Mar 4;10(3):e0118663. doi: 10.1371/journal.pone.0118663 (PMC4349452; doi:10.1371/journal.pone.0118663)
Supplement: S1 Table — Transcript variants uc002atw.1 and uc002atx.1 are translated to M1; uc002aty.1 is translated to M2. Data are presented by mean and SEM. (DOC) [file pone.0118663.s003.doc]

Table S1. Expression and proportion of 14 PKM transcript variants in normal tissue samples.

Transcript variants uc002atw.1 and uc002atx.1 are translated to M1; uc002aty.1 is translated to M2. Data are presented by mean ± SEM.

Table S1. Part A. Expression and proportion of uc002atr.1, uc002ats.1, uc002att.1, uc002atu.1, uc002atv.1, uc002atw.1 and uc002atx.1 in normal tissue samples.

| Tissue types | No. | uc002atr.1 | uc002ats.1 | uc002att.1 | uc002atu.1 | uc002atv.1 | uc002atw.1 | uc002atx.1 |
| --- | --- | --- | --- | --- | --- | --- | --- | --- |
| Bladder Urothelial Carcinoma | 19 | 2134.5±600.5 (6.7±1.8%) | 683.2±122.2 (2.3±0.4%) | 0.0±0.0 (0.0±0.0%) | 496.3±156.1 (1.8±0.5%) | 202.5±24.8 (0.8±0.1%) | 18.2±2.9 (0.1±0.0%) | 2829.2±594.8 (15.3±3.8%) |
| Breast invasive carcinoma | 110 | 774.4±73.4 (6.8±0.8%) | 141.8±23.8 (1.0±0.1%) | 36.0±32.8 (0.1±0.0%) | 378.5±62.7 (2.5±0.3%) | 446.9±46.6 (3.3±0.1%) | 7.6±1.0 (0.1±0.0%) | 1628.9±820.4 (7.8±0.8%) |
| Cervical squamous cell carcinoma and endocervical adenocarcinoma | 3 | 2618.5±1814.9 (14.6±6.8%) | 516.5±193.2 (3.2±0.5%) | 195.7±195.7 (0.8±0.8%) | 447.5±283.9 (2.4±1%) | 146.2±19.6 (1.0±0.2%) | 21.5±2.0 (0.2±0.0%) | 1036.7±784.4 (7.4±4.5%) |
| Colon adenocarcinoma | 41 | 518.4±46.7 (3.3±0.3%) | 356.9±41.6 (2.2±0.2%) | 0.0±0.0 (0.0±0.0%) | 261.3±24.5 (1.6±0.1%) | 115.7±9.1 (0.7±0.0%) | 10.9±1.7 (0.1±0.0%) | 701.7±119.3 (4.4±0.7%) |
| Head and Neck squamous cell carcinoma | 43 | 1425.6±369.8 (4.1±1.3%) | 2878.1±204.6 (6.7±0.4%) | 0.1±0.1 (0.0±0.0%) | 535.9±81.8 (1.4±0.3%) | 478.0±28.2 (1.1±0.0%) | 14.6±1.9 (0.0±0.0%) | 6652.2±2012.7 (13.0±3.6%) |
| Kidney Chromophobe | 25 | 2045.0±286.0 (5.0±0.4%) | 1401.4±156.6 (3.5±0.2%) | 0.0±0.0 (0.0±0.0%) | 521.4±68.2 (1.4±0.2%) | 445.4±37.3 (1.1±0.0%) | 23.7±4.9 (0.1±0.0%) | 674.0±141.6 (1.5±0.3%) |
| Kidney renal clear cell carcinoma | 72 | 1155.6±159.1 (3.6±0.4%) | 322.3±33.2 (0.9±0.1%) | 7.7±3.1 (0.0±0.0%) | 1671.1±219.2 (4.1±0.3%) | 1113.4±92.9 (2.9±0.1%) | 14.8±1.9 (0.0±0.0%) | 61.4±26.3 (0.2±0.1%) |
| Kidney renal papillary cell carcinoma | 30 | 1322.2±179.8 (4.4±0.4%) | 1117.4±154.1 (3.5±0.3%) | 0.2±0.2 (0.0±0.0%) | 402.9±96.1 (1.6±0.4%) | 351.1±39.9 (1.2±0.1%) | 15.1±3 (0.1±0.0%) | 220.3±136.7 (0.4±0.2%) |
| Liver hepatocellular carcinoma | 50 | 303.9±25.6 (23.2±1.6%) | 76.3±11.5 (6.7±1.3%) | 1.3±1.0 (0.1±0.1%) | 9.7±3.7 (0.9±0.3%) | 12.0±2.3 (0.8±0.1%) | 1.9±0.7 (0.1±0.0%) | 10.0±2.5 (0.7±0.2%) |
| Lung adenocarcinoma | 58 | 884.1±95.6 (5.3±0.4%) | 341.2±37.6 (2.1±0.2%) | 0.0±0.0 (0.0±0.0%) | 189.4±23.2 (1.2±0.1%) | 131.3±8.5 (0.8±0.0%) | 15.9±2.0 (0.1±0.0%) | 550.5±36.6 (3.6±0.2%) |
| Lung squamous cell carcinoma | 50 | 873.6±110.0 (4.9±0.6%) | 323.1±41.3 (1.7±0.2%) | 10.3±2.5 (0.0±0.0%) | 1086.3±171.7 (5.1±0.7%) | 258.5±27.5 (1.3±0.1%) | 18.9±2.3 (0.1±0.0%) | 737.0±40.1 (4.1±0.2%) |
| Pancreatic adenocarcinoma | 3 | 1134.8±586.6 (6.4±2.8%) | 552.4±306.7 (3.0±1.5%) | 0.0±0.0 (0.0±0.0%) | 218.2±109.2 (1.6±0.9%) | 128.4±47.2 (0.7±0.2%) | 3.2±3.2 (0.0±0.0%) | 570.5±257.8 (3.9±2%) |
| Prostate adenocarcinoma | 50 | 1404.5±203.6 (7.7±1.1%) | 823±93.8 (4.5±0.5%) | 15.8±7.3 (0.1±0.1%) | 151.6±16.4 (0.9±0.1%) | 144.7±7.3 (0.8±0.0%) | 13.0±1.6 (0.1±0.0%) | 1906.9±242.4 (10.8±1.3%) |
| Rectum adenocarcinoma | 9 | 489.0±120.5 (3.1±0.8%) | 202.6±54.9 (1.2±0.3%) | 0.0±0.0 (0.0±0.0%) | 239.9±62.6 (1.4±0.3%) | 131±8.2 (0.9±0.1%) | 10±5.8 (0.1±0.0%) | 1115.6±187.6 (7.6±1.4%) |
| Sarcoma | 2 | 2638.6±1697.9 (13.0±7.7%) | 574.1±95.6 (3.0±0.7%) | 0.0±0.0 (0.0±0.0%) | 0.0±0.0 (0.0±0.0%) | 41.5±41.5 (0.2±0.2%) | 31.5±3.2 (0.2±0.0%) | 2145.3±2125 (10.3±10.1%) |
| Skin Cutaneous Melanoma | 1 | 4040.8 (8.2%) | 500.9 (1.0%) | 0.0 (0.0%) | 1659.9 (3.4%) | 468.6 (0.9%) | 15.4 (0.0%) | 958.9 (1.9%) |
| Thyroid carcinoma | 29 | 434.3±176.5 (3.5±1.8%) | 355.3±108.5 (2.5±0.7%) | 9.8±6.8 (0.1±0.1%) | 218.3±91.2 (1.4±0.5%) | 123.0±10.6 (0.9±0.1%) | 11.7±2.1 (0.1±0.0%) | 152.4±30.8 (1.1±0.2%) |
| Uterine Corpus Endometrial Carcinoma | 23 | 1188.8±225.7 (8.3±1.4%) | 1347.2±265.6 (10.3±2.1%) | 0.0±0.0 (0.0±0.0%) | 142±50.7 (1.0±0.3%) | 64.4±12.8 (0.6±0.1%) | 37±7.6 (0.3±0.1%) | 714.6±153.1 (6.6±1.4%) |

Table S1. Part B. Expression and proportion of uc002aty.1, uc002atz.1, uc010bit.1, uc010biu.1, uc010uki.1, uc010ukj.1, uc010ukk.1 and summation of all 14 PKM transcript variants in normal tissue samples.

| Tissue types | uc002aty.1 | uc002atz.1 | uc010bit.1 | uc010biu.1 | uc010uki.1 | uc010ukj.1 | uc010ukk.1 | Sum |
| --- | --- | --- | --- | --- | --- | --- | --- | --- |
| Bladder Urothelial Carcinoma | 19738.6±2151.4 (71.5±3.2%) | 37.1±16.5 (0.2±0.1%) | 29.5±7.6 (0.1±0.0%) | 262.8±46.8 (1.1±0.2%) | 14.2±2.9 (0.1±0.0%) | 10.6±3.9 (0.0±0.0%) | 0.0±0.0 (0.0±0.0%) | 26456.5±2131.1 |
| Breast invasive carcinoma | 9389.3±369.4 (73.2±1.2%) | 520.9±30.1 (4.7±0.3%) | 20.0±2.3 (0.2±0.0%) | 71.8±25.1 (0.4±0.1%) | 7.6±1.0 (0.1±0.0%) | 3.6±0.6 (0.0±0.0%) | 0.7±0.5 (0.0±0.0%) | 13428.1±964.3 |
| Cervical squamous cell carcinoma and endocervical adenocarcinoma | 10328.4±2638.6 (66.1±3.6%) | 0.0±0.0 (0.0±0.0%) | 52.0±36.7 (0.6±0.5%) | 495.6±113.9 (3.5±0.8%) | 5.9±3.1 (0.0±0.0%) | 6.7±3.5 (0.1±0.0%) | 0.0±0.0 (0.0±0.0%) | 15871.2±4502.8 |
| Colon adenocarcinoma | 14238.4±464.7 (87.2±1%) | 2.7±1.7 (0.0±0.0%) | 5.4±1.6 (0.0±0.0%) | 92.6±22.6 (0.6±0.1%) | 2.2±0.6 (0.0±0.0%) | 3.4±0.8 (0.0±0.0%) | 0.0±0.0 (0.0±0.0%) | 16309.6±467.5 |
| Head and Neck squamous cell carcinoma | 30628.3±1967.5 (72.2±3.7%) | 50.2±17.0 (0.1±0.1%) | 25.4±4.4 (0.1±0.0%) | 516.1±99.0 (1.2±0.2%) | 21.7±2.6 (0.0±0.0%) | 13.7±1.8 (0.0±0.0%) | 3.6±2.3 (0.0±0.0%) | 43243.5±1801.9 |
| Kidney Chromophobe | 34616.3±2981.1 (86.7±0.5%) | 1.1±0.6 (0.0±0.0%) | 29±5.2 (0.1±0.0%) | 205.6±45.1 (0.6±0.1%) | 20.4±4.1 (0.0±0.0%) | 19.6±5.7 (0.0±0.0%) | 0.5±0.5 (0.0±0.0%) | 40003.4±3497.7 |
| Kidney renal clear cell carcinoma | 30923.9±2191.3 (83.3±0.5%) | 1515.8±121.0 (4.6±0.3%) | 16.5±2.0 (0.0±0.0%) | 106.3±25.4 (0.2±0.0%) | 9.2±1.2 (0.0±0.0%) | 5.0±1.2 (0.0±0.0%) | 0.0±0.0 (0.0±0.0%) | 36923.1±2568.8 |
| Kidney renal papillary cell carcinoma | 26388.7±3077.5 (86.4±0.9%) | 320.0±102.4 (1.5±0.5%) | 22.3±4.6 (0.1±0.0%) | 229.1±38.2 (0.7±0.1%) | 9.9±2.3 (0.0±0.0%) | 3.3±0.9 (0.0±0.0%) | 0.0±0.0 (0.0±0.0%) | 30402.3±3491.7 |
| Liver hepatocellular carcinoma | 851.7±80.2 (57.8±3.0%) | 87.3±15.4 (7.1±1.4%) | 1.1±0.6 (0.1±0.0%) | 31.1±4.2 (2.2±0.2%) | 0.2±0.2 (0.0±0.0%) | 0.3±0.3 (0.0±0.0%) | 2.3±0.9 (0.2±0.1%) | 1389.0±108.6 |
| Lung adenocarcinoma | 13271.6±425.4 (85.6±0.5%) | 14.1±5.3 (0.1±0.0%) | 20.7±3.1 (0.1±0.0%) | 162.5±18.6 (1.0±0.1%) | 7.3±1.1 (0.0±0.0%) | 2.6±0.8 (0.0±0.0%) | 0.0±0.0 (0.0±0.0%) | 15591.2±528.8 |
| Lung squamous cell carcinoma | 14873.1±498.7 (80.9±0.9%) | 209.4±48.5 (1.0±0.2%) | 21.6±2.6 (0.1±0.0%) | 110±23.4 (0.6±0.1%) | 9.7±1.4 (0.1±0.0%) | 4.8±0.9 (0.0±0.0%) | 0.0±0.0 (0.0±0.0%) | 18536.1±690.8 |
| Pancreatic adenocarcinoma | 13797.6±2390.7 (83.2±2.8%) | 14.7±14.7 (0.1±0.1%) | 56.5±49.6 (0.3±0.3%) | 149.1±123.6 (0.8±0.6%) | 10.4±0.9 (0.1±0.0%) | 3.4±3.4 (0.0±0.0%) | 0.0±0.0 (0.0±0.0%) | 16639.1±2892.3 |
| Prostate adenocarcinoma | 13390.6±578.2 (73.4±1.7%) | 30.2±13.0 (0.2±0.1%) | 31.8±3.6 (0.2±0.0%) | 239.4±43.0 (1.3±0.2%) | 8.6±1.8 (0.0±0.0%) | 5.6±1.1 (0.0±0.0%) | 0.7±0.4 (0.0±0.0%) | 18166.4±592.3 |
| Rectum adenocarcinoma | 13610.0±1445.1 (84.5±1.6%) | 1.2±1.2 (0.0±0.0%) | 11.2±8.9 (0.1±0.1%) | 117.5±71.2 (1.0±0.7%) | 2.7±1.4 (0.0±0.0%) | 4.1±1.4 (0.0±0.0%) | 0.0±0.0 (0.0±0.0%) | 15934.8±1458.1 |
| Sarcoma | 12499.5±3080.5 (66.5±21.5%) | 223.9±78.8 (1.1±0.3%) | 39.1±39.1 (0.2±0.2%) | 1114.2±894.6 (5.4±4.2%) | 0.0±0.0 (0.0±0.0%) | 0.0±0.0 (0.0±0.0%) | 0.0±0.0 (0.0±0.0%) | 19307.7±1614.4 |
| Skin Cutaneous Melanoma | 41121.8 (83.1%) | 0.0 (0.0%) | 13.1(0.0%) | 656.6 (1.3%) | 28.9 (0.1%) | 8.4 (0.0%) | 0.0 (0.0%) | 49473.3 |
| Thyroid carcinoma | 12224.6±463.1 (89.2±2.6%) | 33.0±22.1 (0.3±0.2%) | 38.7±6.3 (0.3±0.0%) | 58.0±14.0 (0.5±0.1%) | 11.8±1.7 (0.1±0.0%) | 4.1±0.9 (0.0±0.0%) | 0.0±0.0 (0.0±0.0%) | 13674.9±424.1 |
| Uterine Corpus Endometrial Carcinoma | 8703.9±823.5 (66.4±2.7%) | 125.1±33.9 (1±0.3%) | 47.1±12.5 (0.5±0.2%) | 537.4±92.6 (4.9±1.1%) | 5.5±1.5 (0.0±0.0%) | 2.4±1.0 (0.0±0.0%) | 3.8±3.8 (0.0±0.0%) | 12919.1±1018.1 |
