# Supplementary material for: Isoform Switch of Pyruvate Kinase M1 Indeed Occurs but Not to Pyruvate Kinase M2 in Human Tumorigenesis
Source: PLoS One. 2015 Mar 4;10(3):e0118663. doi: 10.1371/journal.pone.0118663 (PMC4349452; doi:10.1371/journal.pone.0118663)
Supplement: S2 Table — (DOC) [file pone.0118663.s004.doc]

Table S2. Expression and proportion of 14 PKM transcript variants in tumor tissue samples. Transcript variants uc002atw.1 and uc002atx.1 are translated to M1; uc002aty.1 is translated to M2. Data are presented by mean and SEM.

Table S2. Part A. Expression and proportion of uc002atr.1, uc002ats.1, uc002att.1, uc002atu.1, uc002atv.1, uc002atw.1 and uc002atx.1 in tumor tissue samples.

| Tissue types | No. | uc002atr.1 | uc002ats.1 | uc002att.1 | uc002atu.1 | uc002atv.1 | uc002atw.1 | uc002atx.1 |
| --- | --- | --- | --- | --- | --- | --- | --- | --- |
| Acute Myeloid Leukemia | 173 | 286.5±50.5 (2.1±0.3%) | 375.9±40.4 (2.8±0.2%) | 25.8±7.7 (0.2±0.0%) | 852.0±49.7 (6.7±0.3%) | 153.1±8.7 (1.2±0.0%) | 50.3±3.9 (0.4±0.0%) | 168.3±21.5 (1.7±0.2%) |
| Adrenocortical carcinoma | 79 | 6191.6±734.2 (21.1±2.3%) | 2548.4±240.9 (7.8±0.5%) | 95.6±22.8 (0.4±0.1%) | 701.4±78.8 (2.4±0.3%) | 207.9±19.8 (0.6±0.0%) | 13.4±1.6 (0.0±0.0%) | 348.9±109.6 (1.1±0.3%) |
| Bladder Urothelial Carcinoma | 241 | 6350.4±393.9 (16.7±1.0%) | 2002.4±121.9 (5.1±0.3%) | 10.2±4.9 (0.0±0.0%) | 834.8±108.4 (1.9±0.1%) | 328.1±29.5 (0.7±0.0%) | 18.4±1.1 (0.1±0.0%) | 517.6±113.9 (1.1±0.2%) |
| Brain Lower Grade Glioma | 469 | 645.2±52.6 (3.0±0.3%) | 385.3±29.9 (1.8±0.1%) | 4.4±2.3 (0.0±0.0%) | 339.8±23.3 (1.6±0.1%) | 282.4±7.1 (1.3±0.0%) | 46.3±1.3 (0.2±0.0%) | 4074.5±232.1 (17.6±0.8%) |
| Breast invasive carcinoma | 1044 | 2463.6±98.7 (10.3±0.4%) | 524.0±26.8 (2.1±0.1%) | 7.1±1.1 (0.0±0.0%) | 1054.6±41.4 (4.0±0.1%) | 635.7±13.6 (2.5±0.0%) | 18.4±1.4 (0.1±0.0%) | 199.1±13.5 (0.9±0.1%) |
| Cervical squamous cell carcinoma and endocervical adenocarcinoma | 186 | 7198.2±571.0 (17.0±1.4%) | 1574.9±123.2 (3.6±0.2%) | 6.8±3.6 (0.0±0.0%) | 848.0±76.3 (1.9±0.2%) | 356.8±24.2 (0.7±0.0%) | 17.3±1.3 (0.0±0.0%) | 265.0±31.4 (0.6±0.1%) |
| Colon adenocarcinoma | 262 | 2456.4±140.3 (8.1±0.3%) | 920.0±54.2 (3.1±0.2%) | 0.0±0.0 (0.0±0.0%) | 475.7±27.1 (1.6±0.1%) | 256.9±9.1 (0.8±0.0%) | 12.1±0.8 (0.0±0.0%) | 182.5±19.8 (0.7±0.1%) |
| Glioblastoma multiforme | 169 | 1374.9±127.0 (4.0±0.3%) | 2114.0±100.6 (6.4±0.2%) | 5.0±3.1 (0.0±0.0%) | 327.1±20.7 (0.9±0.0%) | 153.0±5.9 (0.5±0.0%) | 59.4±2.4 (0.2±0.0%) | 1788.2±157.8 (5.9±0.6%) |
| Head and Neck squamous cell carcinoma | 498 | 3248.6±281.5 (5.5±0.5%) | 4804.7±125.6 (7.3±0.1%) | 2.7±0.8 (0.0±0.0%) | 1158.8±88.2 (1.7±0.1%) | 788.4±26.9 (1.1±0.0%) | 26.1±1.3 (0.0±0.0%) | 1299.1±93.9 (2.1±0.2%) |
| Kidney Chromophobe | 66 | 5331.7±646.1 (11.5±1.7%) | 3351.2±310.8 (6.6±0.5%) | 5.9±3.8 (0.0±0.0%) | 1657.5±193.6 (3.3±0.4%) | 424.0±24.8 (0.8±0.0%) | 23.1±3.8 (0.0±0.0%) | 54.1±18.0 (0.1±0.0%) |
| Kidney renal clear cell carcinoma | 519 | 5558.8±360.9 (11.3±0.7%) | 1013.6±67.8 (1.9±0.1%) | 19.5±3.1 (0.0±0.0%) | 3818±126.6 (7.0±0.2%) | 1104.2±23.4 (2.1±0.0%) | 33.7±1.4 (0.1±0.0%) | 78.8±9.7 (0.2±0.0%) |
| Kidney renal papillary cell carcinoma | 198 | 8174.6±695.5 (17.0±1.4%) | 2724.6±225.2 (5.4±0.4%) | 8.6±3.4 (0.0±0.0%) | 956±85.9 (2.0±0.2%) | 393.8±20.3 (0.8±0.0%) | 17.0±1.2 (0.0±0.0%) | 136.6±23.1 (0.2±0.0%) |
| Liver hepatocellular carcinoma | 191 | 1306.2±226.4 (22.1±1.3%) | 307.4±42.0 (6.0±0.5%) | 3.0±1.1 (0.1±0.0%) | 185.9±38.2 (2.6±0.3%) | 49.7±8.4 (0.6±0.0%) | 4.0±0.6 (0.1±0.0%) | 50.2±17.8 (0.9±0.1%) |
| Lung adenocarcinoma | 490 | 2355.3±124.8 (7.4±0.4%) | 876.6±40.2 (2.9±0.1%) | 2.3±1.1 (0.0±0.0%) | 569.6±39.8 (1.7±0.1%) | 304.2±11.5 (0.9±0.0%) | 14.1±0.7 (0.0±0.0%) | 441.3±28.6 (1.4±0.1%) |
| Lung squamous cell carcinoma | 490 | 2750.0±147.6 (5.7±0.3%) | 1088.3±49.8 (2.3±0.1%) | 46.9±5.6 (0.1±0.0%) | 3407.3±181.4 (6.3±0.3%) | 776.2±25.9 (1.5±0.0%) | 22.6±0.9 (0.0±0.0%) | 849.2±56.2 (1.8±0.1%) |
| Lymphoid Neoplasm Diffuse Large B-cell Lymphoma | 28 | 13597.2±1697.3 (26.2±3.1%) | 2696.0±331.7 (5.1±0.6%) | 48.3±31.1 (0.2±0.1%) | 4640.6±506.9 (8.6±0.7%) | 262.6±36.2 (0.4±0.1%) | 32.0±5.3 (0.1±0.0%) | 2426.4±400.9 (4.3±0.7%) |
| Ovarian serous cystadenocarcinoma | 266 | 2061.8±130.7 (4.1±0.2%) | 10558.8±397.0 (21.7±0.3%) | 617.1±29.8 (1.3±0.0%) | 233.7±26.6 (0.5±0.0%) | 163.9±7.0 (0.4±0.0%) | 105.9±3.8 (0.2±0.0%) | 1187.5±117.6 (2.5±0.2%) |
| Pancreatic adenocarcinoma | 85 | 2872.1±413.6 (8.7±1.2%) | 1103.5±120.0 (3.5±0.4%) | 0.1±0.1 (0.0±0.0%) | 456.6±53.0 (1.5±0.1%) | 283.7±18.7 (0.9±0.0%) | 18.5±1.8 (0.1±0.0%) | 930.5±105.8 (2.9±0.3%) |
| Prostate adenocarcinoma | 333 | 1663.4±100.6 (11.2±0.7%) | 651.7±28.1 (4.3±0.2%) | 8.7±2.7 (0.1±0.0%) | 353.7±23.8 (2.3±0.1%) | 104.8±3.5 (0.7±0.0%) | 11.2±0.7 (0.1±0.0%) | 630.8±38.9 (4.2±0.2%) |
| Rectum adenocarcinoma | 164 | 1816.0±178.0 (7.9±0.8%) | 2313.0±229.2 (9.5±0.7%) | 0.5±0.5 (0.0±0.0%) | 560.1±66.5 (2.3±0.2%) | 124.7±10.1 (0.5±0.0%) | 44.9±4.4 (0.2±0.0%) | 108.5±18.2 (0.5±0.1%) |
| Sarcoma | 105 | 2654.9±431.8 (10.8±1.5%) | 1053.8±111.3 (4.3±0.4%) | 46.2±30.2 (0.2±0.1%) | 166.5±25.7 (0.6±0.1%) | 214.9±18.0 (0.9±0.1%) | 32.4±3.0 (0.2±0.0%) | 2960.7±392.9 (10.7±1.1%) |
| Skin Cutaneous Melanoma | 373 | 3940.0±223.4 (8.0±0.5%) | 1867.3±86.9 (3.7±0.1%) | 15.4±5.4 (0.0±0.0%) | 659.8±43.2 (1.4±0.1%) | 553.3±18.5 (1.1±0.0%) | 38.9±2.0 (0.1±0.0%) | 1631.8±129.8 (3.1±0.2%) |
| Thyroid carcinoma | 275 | 1652.7±145.9 (8.6±0.8%) | 727.9±43.8 (3.6±0.2%) | 15.7±4.4 (0.1±0.0%) | 254.6±23.8 (1.2±0.1%) | 197.3±6.8 (1.0±0.0%) | 11.5±0.7 (0.1±0.0%) | 256.2±18.3 (1.2±0.1%) |
| Uterine Carcinosarcoma | 57 | 4853.7±642.0 (16.5±2.1%) | 2679.7±266.2 (8.4±0.7%) | 38.5±20.1 (0.1±0.1%) | 775.1±107.7 (2.5±0.4%) | 220.7±19.8 (0.7±0.0%) | 21.7±2.6 (0.1±0.0%) | 954.5±195.3 (2.9±0.5%) |
| Uterine Corpus Endometrial Carcinoma | 529 | 9206±362.8 (27.6±1%) | 4989.6±163.5 (13.5±0.3%) | 4.9±2.3 (0.0±0.0%) | 286.4±31.3 (0.7±0.1%) | 91.1±5.8 (0.2±0.0%) | 61.9±3.3 (0.2±0.0%) | 94.7±22.0 (0.3±0.1%) |

Table S2. Part B. Expression and proportion of uc002aty.1, uc002atz.1, uc010bit.1, uc010biu.1, uc010uki.1, uc010ukj.1, uc010ukk.1 and summation of all 14 PKM transcript variants in tumor tissue samples.

| Tissue types | uc002aty.1 | uc002atz.1 | uc010bit.1 | uc010biu.1 | uc010uki.1 | uc010ukj.1 | uc010ukk.1 | Sum |
| --- | --- | --- | --- | --- | --- | --- | --- | --- |
| Acute Myeloid Leukemia | 10138.9±397.6 (81.3±0.5%) | 73.9±16.8 (0.7±0.1%) | 37.1±4.1 (0.3±0.0%) | 268.9±20.9 (2.3±0.2%) | 12.4±1.7 (0.1±0.0%) | 9.1±1.3 (0.1±0.0%) | 21.7±2.4 (0.2±0.0%) | 12474.0±486.7 |
| Adrenocortical carcinoma | 21651.2±1652.8 (65.1±2.6%) | 94.9±40.9 (0.3±0.1%) | 17.4±2.3 (0.1±0.0%) | 318.3±31.6 (1±0.1%) | 14.1±1.7 (0.0±0.0%) | 8.9±1.9 (0.0±0.0%) | 2.1±1.1 (0.0±0.0%) | 32214.1±1974.6 |
| Bladder Urothelial Carcinoma | 31234.3±1323.4 (73±1.1%) | 110.2±18.1 (0.3±0.1%) | 37.4±3.2 (0.1±0.0%) | 307.5±16.5 (0.8±0.0%) | 18.2±1.6 (0.0±0.0%) | 14.6±1.1 (0.0±0.0%) | 3.5±1.3 (0.0±0.0%) | 41787.6±1582.4 |
| Brain Lower Grade Glioma | 15196.9±430.8 (71.7±0.8%) | 20.1±2.5 (0.1±0.0%) | 67.4±2.6 (0.4±0.0%) | 431.3±19.7 (2.0±0.1%) | 40.8±1.3 (0.2±0.0%) | 9.7±0.8 (0.0±0.0%) | 0.7±0.3 (0.0±0.0%) | 21544.6±490.3 |
| Breast invasive carcinoma | 19295.0±309.8 (76.0±0.4%) | 849.4±27.4 (3.6±0.1%) | 22.1±0.8 (0.1±0.0%) | 82.1±4.5 (0.4±0.0%) | 10.5±0.4 (0.0±0.0%) | 6.6±0.3 (0.0±0.0%) | 2.3±0.6 (0.0±0.0%) | 25170.7±369.7 |
| Cervical squamous cell carcinoma and endocervical adenocarcinoma | 35195.5±1480.4 (74.7±1.5%) | 171.2±30.2 (0.5±0.1%) | 38.8±2.8 (0.1±0.0%) | 360.5±23.6 (0.8±0.0%) | 21.3±1.9 (0.0±0.0%) | 17.6±1.4 (0.0±0.0%) | 8.6±3.2 (0.0±0.0%) | 46080.3±1610.2 |
| Colon adenocarcinoma | 25747.7±742.9 (84.7±0.4%) | 1.4±0.6 (0.0±0.0%) | 18.9±1.4 (0.1±0.0%) | 208.6±11.4 (0.7±0.0%) | 10.9±0.8 (0.0±0.0%) | 10.6±0.9 (0.0±0.0%) | 0.2±0.1 (0.0±0.0%) | 30302.0±845.9 |
| Glioblastoma multiforme | 26501.9±898.7 (80.1±0.7%) | 59.1±8.5 (0.2±0.0%) | 34.9±3.3 (0.1±0.0%) | 493.7±35.0 (1.6±0.1%) | 28.6±1.7 (0.1±0.0%) | 7.5±0.8 (0.0±0.0%) | 2.8±1.6 (0.0±0.0%) | 32950.2±1063.5 |
| Head and Neck squamous cell carcinoma | 55266.4±1310.9 (80.9±0.6%) | 275.9±47.4 (0.4±0.1%) | 45.1±1.9 (0.1±0.0%) | 507.9±24.1 (0.8±0.0%) | 28.7±1.0 (0.0±0.0%) | 28.0±1.1 (0.0±0.0%) | 2.8±0.7 (0.0±0.0%) | 67483.1±1452.2 |
| Kidney Chromophobe | 38126.8±1647.2 (76.6±2.2%) | 55.6±33.9 (0.2±0.1%) | 20.4±3.7 (0.0±0.0%) | 352.8±35.6 (0.7±0.1%) | 30.2±2.9 (0.1±0.0%) | 22.1±2.7 (0.0±0.0%) | 0.0±0.0 (0.0±0.0%) | 49455.4±1663.2 |
| Kidney renal clear cell carcinoma | 39704.3±743.7 (74.0±0.8%) | 1425.5±58.2 (2.9±0.1%) | 39.0±1.8 (0.1±0.0%) | 126.3±9.5 (0.3±0.1%) | 24.5±0.9 (0.0±0.0%) | 18.5±0.9 (0.0±0.0%) | 15.9±7.5 (0.0±0.0%) | 52980.5±823.6 |
| Kidney renal papillary cell carcinoma | 37952.0±1370.1 (73.1±1.6%) | 293.0±44.6 (0.7±0.1%) | 30.5±2.5 (0.1±0.0%) | 268.3±16.8 (0.5±0.0%) | 13.0±1.0 (0.0±0.0%) | 10.6±1.1 (0.0±0.0%) | 3.1±1.2 (0.0±0.0%) | 50981.8±1409.6 |
| Liver hepatocellular carcinoma | 4439.3±652.8 (63.8±1.6%) | 67.1±12.0 (2.2±0.4%) | 4.1±1.0 (0.0±0.0%) | 78.4±22.2 (1.2±0.1%) | 1.8±0.4 (0.0±0.0%) | 3.0±0.7 (0.0±0.0%) | 1.5±0.5 (0.1±0.0%) | 6501.4±868.7 |
| Lung adenocarcinoma | 27611.2±639.9 (84.7±0.4%) | 57.3±9.9 (0.2±0.0%) | 26.3±1.4 (0.1±0.0%) | 183.1±12.1 (0.6±0.1%) | 12.8±0.7 (0.0±0.0%) | 11.1±0.7 (0.0±0.0%) | 0.4±0.1 (0.0±0.0%) | 32465.6±723.3 |
| Lung squamous cell carcinoma | 40878.9±859.6 (80.8±0.4%) | 384.6±51.2 (0.7±0.1%) | 49.9±2.1 (0.1±0.0%) | 309.3±26.6 (0.6±0.1%) | 26.6±1.1 (0.1±0.0%) | 18.1±1.0 (0.0±0.0%) | 2.4±0.7 (0.0±0.0%) | 50610.2±1053.1 |
| Lymphoid Neoplasm Diffuse Large B-cell Lymphoma | 29964.0±3077.2 (53.0±3.8%) | 73.0±47.9 (0.2±0.1%) | 33.0±10.1 (0.1±0.0%) | 967.3±161.5 (1.7±0.2%) | 10.7±2.0 (0.0±0.0%) | 15.2±3.9 (0.0±0.0%) | 9.6±6.6 (0.0±0.0%) | 54775.9±4312.1 |
| Ovarian serous cystadenocarcinoma | 32862.0±1039.5 (69.1±0.5%) | 11.4±3.5 (0.0±0.0%) | 44.0±2.9 (0.1±0.0%) | 3.3±1.0 (0.0±0.0%) | 20.7±1.3 (0.0±0.0%) | 21.8±1.4 (0.0±0.0%) | 6.3±1.0 (0.0±0.0%) | 47898.4±1523.4 |
| Pancreatic adenocarcinoma | 27044.0±1658.0 (81.5±1.4%) | 60.9±20.8 (0.2±0.1%) | 30.8±3.6 (0.1±0.0%) | 196.8±24.2 (0.6±0.1%) | 12.6±1.7 (0.0±0.0%) | 11.6±1.7 (0.0±0.0%) | 0.3±0.3 (0.0±0.0%) | 33022.1±1845.3 |
| Prostate adenocarcinoma | 11540.6±233.8 (75.8±0.9%) | 21.2±5.6 (0.2±0.0%) | 26.9±1.7 (0.2±0.0%) | 128.2±9.0 (0.8±0.1%) | 4.6±0.4 (0.0±0.0%) | 2.8±0.3 (0.0±0.0%) | 1.4±0.5 (0.0±0.0%) | 15149.9±242.5 |
| Rectum adenocarcinoma | 18572.1±704.6 (75.8±1.2%) | 211.5±36.8 (1±0.2%) | 17.2±1.9 (0.1±0.0%) | 609.0±164.0 (2.1±0.2%) | 7.8±0.9 (0.0±0.0%) | 6.7±0.8 (0.0±0.0%) | 22.9±22.7 (0.1±0.1%) | 24415.0±913.7 |
| Sarcoma | 16233.0±1090.8 (69.3±1.9%) | 120.0±27.3 (0.5±0.1%) | 31.4±3.5 (0.2±0.0%) | 555.9±57.5 (2.2±0.2%) | 11.0±1.4 (0.0±0.0%) | 7.6±1.4 (0.0±0.0%) | 6.1±5.6 (0.0±0.0%) | 24094.6±1487.6 |
| Skin Cutaneous Melanoma | 41305.3±1119.8 (81.2±0.6%) | 72.4±20.2 (0.2±0.0%) | 44.1±6.3 (0.1±0.0%) | 524.6±31.7 (1.1±0.1%) | 31.2±1.4 (0.1±0.0%) | 15.5±0.9 (0.0±0.0%) | 2.3±1.0 (0.0±0.0%) | 50701.8±1333.2 |
| Thyroid carcinoma | 16598.0±403.4 (83.3±0.9%) | 48.2±15.6 (0.3±0.1%) | 30.9±1.9 (0.2±0.0%) | 93.4±6.8 (0.4±0.0%) | 7.9±0.5 (0.0±0.0%) | 3.7±0.4 (0.0±0.0%) | 2.6±0.9 (0.0±0.0%) | 19900.7±444.0 |
| Uterine Carcinosarcoma | 21672.4±1577.2 (67.3±2.7%) | 86.2±32.1 (0.4±0.1%) | 24.1±4.0 (0.1±0.0%) | 316.7±32.0 (1.0±0.1%) | 17.8±3.1 (0.1±0.0%) | 10.2±1.7 (0.0±0.0%) | 3.4±2.2 (0.0±0.0%) | 31674.7±1783.1 |
| Uterine Corpus Endometrial Carcinoma | 20109.9±602.3 (51.9±1.0%) | 1386.7±95.2 (3.7±0.2%) | 27.3±1.9 (0.1±0.0%) | 541.5±30.7 (1.5±0.1%) | 9.1±0.8 (0.0±0.0%) | 9.7±0.7 (0.0±0.0%) | 93.7±37.7 (0.2±0.1%) | 36912.6±779.6 |
