# Supplementary material for: Isoform Switch of Pyruvate Kinase M1 Indeed Occurs but Not to Pyruvate Kinase M2 in Human Tumorigenesis
Source: PLoS One. 2015 Mar 4;10(3):e0118663. doi: 10.1371/journal.pone.0118663 (PMC4349452; doi:10.1371/journal.pone.0118663)
Supplement: S3 Table — (DOC) [file pone.0118663.s005.doc]

Table S3. Expression and proportion of PKM1, PKM2 and PKM All data are presented by mean and SEM

| Tissue Types | Case No. | |  | | M1 | |  | | M2 | |  | | Sum | |  |
| --- | --- | --- | --- | --- | --- | --- | --- | --- | --- | --- | --- | --- | --- | --- | --- |
|  | Normal | Tumor | | Normal | | Tumor | | Normal | | Tumor | | Normal | | Tumor | |
| LAML | 0 | 173 | | N/A | | 218.6±22.8 (2.1±0.2%) | | N/A | | 10138.9±397.6 (81.3±0.5%) | | N/A | | 12474.0±486.7 | |
| ACC | 0 | 79 | | N/A | | 362.3±109.8 (1.2±0.3%) | | N/A | | 21651.2±1652.8 (65.1±2.6%) | | N/A | | 32214.1±1974.6 | |
| BLCA | 19 | 241 | | 2847.3±594.8 (15.4±3.9%) | | 536.0±114.0 (1.1±0.2%) | | 19738.6±2151.4 (71.5±3.2%) | | 31234.3±1323.4 (73.0±1.1%) | | 26456.5±2131.1 | | 41787.6±1582.4 | |
| LGG | 0 | 469 | | N/A | | 4120.8±232.2 (17.8±0.8%) | | N/A | | 15196.9±430.8 (71.7±0.8%) | | N/A | | 21544.6±490.3 | |
| BRCA | 110 | 1044 | | 1636.5±820.4 (7.8±0.8%) | | 217.5±13.6 (1.0±0.1%) | | 9389.3±369.4 (73.2±1.2%) | | 19295.0±309.8 (76.0±0.4%) | | 13428.1±964.3 | | 25170.7±369.7 | |
| CESC | 3 | 186 | | 1058.2±783.3 (7.5±4.5%) | | 282.2±31.5 (0.6±0.1%) | | 10328.4±2638.6 (66.1±3.6%) | | 35195.5±1480.4 (74.7±1.5%) | | 15871.2±4502.8 | | 46080.3±1610.2 | |
| COAD | 41 | 262 | | 712.6±119.2 (4.4±0.7%) | | 194.6±20.0 (0.8±0.1%) | | 14238.4±464.7 (87.2±1.0%) | | 25747.7±742.9 (84.7±0.4%) | | 16309.6±467.5 | | 30302.0±845.9 | |
| GBM | 0 | 169 | | N/A | | 1847.7±157.9 (6.1±0.6%) | | N/A | | 26501.9±898.7 (80.1±0.7%) | | N/A | | 32950.2±1063.5 | |
| HNSC | 43 | 498 | | 6666.8±2012.3 (13.0±3.6%) | | 1325.2±94.1 (2.1±0.2%) | | 30628.3±1967.5 (72.2±3.7%) | | 55266.4±1310.9 (80.9±0.6%) | | 43243.5±1801.9 | | 67483.1±1452.2 | |
| KICH | 25 | 66 | | 697.7±142.8 (1.5±0.3%) | | 77.1±18.8 (0.1±0.0%) | | 34616.3±2981.1 (86.7±0.5%) | | 38126.8±1647.2 (76.6±2.2%) | | 40003.4±3497.7 | | 49455.4±1663.2 | |
| KIRC | 72 | 519 | | 76.2±26.2 (0.2±0.1%) | | 112.6±9.8 (0.2±0.0%) | | 30923.9±2191.3 (83.3±0.5%) | | 39704.3±743.7 (74.0±0.8%) | | 36923.1±2568.8 | | 52980.5±823.6 | |
| KIRP | 30 | 198 | | 235.4±138.0 (0.4±0.1%) | | 153.6±23.4 (0.3±0.0%) | | 26388.7±3077.5 (86.4±0.9%) | | 37952.0±1370.1 (73.1±1.6%) | | 30402.3±3491.7 | | 50981.8±1409.6 | |
| LIHC | 50 | 191 | | 11.9±2.6 (0.8±0.2%) | | 54.1±17.9 (1.0±0.1%) | | 851.7±80.2 (57.8±3.0%) | | 4439.3±652.8 (63.8±1.6%) | | 1389.0±108.6 | | 6501.4±868.7 | |
| LUAD | 58 | 490 | | 566.4±36.8 (3.7±0.2%) | | 455.5±28.6 (1.5±0.1%) | | 13271.6±425.4 (85.6±0.5%) | | 27611.2±639.9 (84.7±0.4%) | | 15591.2±528.8 | | 32465.6±723.3 | |
| LUSC | 50 | 490 | | 756±40.1 (4.2±0.2%) | | 871.8±56.3 (1.8±0.1%) | | 14873.1±498.7 (80.9±0.9%) | | 40878.9±859.6 (80.8±0.4%) | | 18536.1±690.8 | | 50610.2±1053.1 | |
| DLBC | 0 | 28 | | N/A | | 2458.4±402.1 (4.3±0.7%) | | N/A | | 29964.0±3077.2 (53.0±3.8%) | | N/A | | 54775.9±4312.1 | |
| OV | 0 | 266 | | N/A | | 1293.4±118.5 (2.7±0.2%) | | N/A | | 32862.0±1039.5 (69.1±0.5%) | | N/A | | 47898.4±1523.4 | |
| PAAD | 3 | 85 | | 573.7±259.8 (4.0±2.0%) | | 949.0±106.1 (3.0±0.3%) | | 13797.6±2390.7 (83.2±2.8%) | | 27044.0±1658.0 (81.5±1.4%) | | 16639.1±2892.3 | | 33022.1±1845.3 | |
| PRAD | 50 | 333 | | 1919.9±242.6 (10.9±1.3%) | | 641.9±39.0 (4.3±0.2%) | | 13390.6±578.2 (73.4±1.7%) | | 11540.6±233.8 (75.8±0.9%) | | 18166.4±592.3 | | 15149.9±242.5 | |
| READ | 9 | 164 | | 1125.5±191.6 (7.7±1.4%) | | 153.4±17.7 (0.7±0.1%) | | 13610.0±1445.1 (84.5±1.6%) | | 18572.1±704.6 (75.8±1.2%) | | 15934.8±1458.1 | | 24415.0±913.7 | |
| SARC | 2 | 105 | | 2176.8±2121.7 (10.4±10.1%) | | 2993.1±392.8 (10.9±1.1%) | | 12499.5±3080.5 (66.5±21.5%) | | 16233.0±1090.8 (69.3±1.9%) | | 19307.7±1614.4 | | 24094.6±1487.6 | |
| SKCM | 1 | 373 | | 974.4 (2.0%) | | 1670.7±130.6 (3.1±0.2%) | | 41121.8 (83.1%) | | 41305.3±1119.8 (81.2±0.6%) | | 49473.3 | | 50701.8±1333.2 | |
| THCA | 29 | 275 | | 164.1±30.8 (1.2±0.2%) | | 267.8±18.4 (1.3±0.1%) | | 12224.6±463.1 (89.2±2.6%) | | 16598.0±403.4 (83.3±0.9%) | | 13674.9±424.1 | | 19900.7±444.0 | |
| UCS | 0 | 57 | | N/A | | 976.2±195.8 (2.9±0.5%) | | N/A | | 21672.4±1577.2 (67.3±2.7%) | | N/A | | 31674.7±1783.1 | |
| UCES | 23 | 529 | | 751.6±152.4 (6.9±1.4%) | | 156.6±22.1 (0.4±0.1%) | | 8703.9±823.5 (66.4±2.7%) | | 20109.9±602.3 (51.9±1.0%) | | 12919.1±1018.1 | | 36912.6±779.6 | |
